# Long-Term Cultures of Spinal Cord Interneurons

**DOI:** 10.3389/fncel.2022.827628

**Published:** 2022-02-07

**Authors:** Ingrid Vargova, Jan Kriska, Jessica C. F. Kwok, James W. Fawcett, Pavla Jendelova

**Affiliations:** ^1^Department of Neuroregeneration, Institute of Experimental Medicine, Czech Academy of Sciences, Prague, Czechia; ^2^Second Faculty of Medicine, Charles University, Prague, Czechia; ^3^Department of Cellular Neurophysiology, Institute of Experimental Medicine, Czech Academy of Sciences, Prague, Czechia; ^4^The Center for Reconstructive Neuroscience, Institute of Experimental Medicine, Czech Academy of Sciences, Prague, Czechia; ^5^Faculty of Biological Sciences, University of Leeds, Leeds, United Kingdom; ^6^John van Geest Centre for Brain Repair, Department of Clinical Neurosciences, University of Cambridge, Cambridge, United Kingdom

**Keywords:** spinal interneurons, culture, maturation, axon regeneration, laser axotomy

## Abstract

Spinal cord interneurons (SpINs) are highly diverse population of neurons that play a significant role in circuit reorganization and spontaneous recovery after spinal cord injury. Regeneration of SpIN axons across rodent spinal injuries has been demonstrated after modification of the environment and neurotrophin treatment, but development of methods to enhance the intrinsic regenerative ability of SpINs is needed. There is a lack of described *in vitro* models of spinal cord neurons in which to develop new regeneration treatments. For this reason, we developed a new model of mouse primary spinal cord neuronal culture in which to analyze maturation, morphology, physiology, connectivity and regeneration of identified interneurons. Isolated from E14 mice, the neurons mature over 15 days *in vitro*, demonstrated by expression of maturity markers, electrophysiological patch-clamp recordings, and formation of synapses. The neurons express markers of SpINs, including Tlx3, Lmx1b, Lbx1, Chx10, and Pax2. The neurons demonstrate distinct morphologies and some form perineuronal nets in long-term cultivation. Live neurons in various maturation stages were axotomized, using a 900 nm multiphoton laser and their fate was observed overnight. The percentage of axons that regenerated declined with neuronal maturity. This model of SpINs will be a valuable tool in future regenerative, developmental, and functional studies alongside existing models using cortical or hippocampal neurons.

## Introduction

The intrinsic regeneration capacity of mature mammalian central nervous system (CNS) is poor. This makes spinal cord injury (SCI) a detrimental condition that represents one of the major causes of disability, and treatment possibilities are limited. However, continued research in the field has led to an increased understanding of the causes of the regeneration failure, which if appropriately modulated, can be used for treatment of the SCI. The extracellular environment of the CNS is not favorable for axon outgrowth due to production of growth-inhibiting molecules such as NogoA and CSPGs from glial scars surrounding the damaged tissue (Shen et al., 2009; Schwab and Strittmatter, 2014; Uyeda and Muramatsu, 2020), and there is a lack of some necessary growth factors that provide trophic support for neurons and act as chemoattractants for axons (Blesch et al., 2012; Anderson et al., 2018). Another important limiting factor is the intrinsic loss of regenerative ability in CNS neurons that comes with neuronal maturation. Various factors contribute to this loss of regeneration, including failure of CNS neurons to activate appropriate transcriptional, translational and epigenetic programs at appropriate subcellular locations that would enable axon growth after injury (van Niekerk et al., 2016; Petrova et al., 2021), changes in axonal transport that exclude growth-related molecules from mature axons and decreased signaling in some pathways. A high level of intrinsic regeneration ability is present in immature neurons but ceases abruptly with maturation (Nicholls and Saunders, 1996; Lu et al., 2014; Koseki et al., 2017).

Maturation and aging in the CNS involve complex and numerous pathways, so it is challenging to study their effect on the regeneration ability of neurons *in vivo*. *In vitro* models, on the other hand, can offer a wide variety of tools to study individual neuronal cell types, to regulate and describe neuronal behavior, and to uncover molecular pathways relating to development and regeneration of neurons (Abu-Rub et al., 2010; Franssen et al., 2015). Therefore, describing axon growth inhibition mechanisms *in vitro* is a valuable approach that could lead to further comprehension of the limits of CNS regeneration and subsequently to discovery of new therapeutic avenues. Various *in vitro* models have been established to explore CNS axon regeneration. These include primary cell cultures created by dissociating neural tissue from animals at various ages (Donaldson and Höke, 2014). Dorsal root ganglia (DRGs) (Cheah et al., 2016), hippocampal (Kaech and Banker, 2006; Moore et al., 2009), and cortical cultures (Koseki et al., 2017) are among the most commonly used models. Primary spinal cord cultures have been described as well (Thomson et al., 2008; Eldeiry et al., 2017), but their application in axon regeneration studies has so far been limited. Investigation of the regenerative capacity of spinal interneurons (SpINs) is of particular interest, as it was shown, that less severe, anatomically incomplete SCI can result in partial recovery that follows after spontaneous reorganization of neural circuits (Bareyre et al., 2004; Martinez et al., 2012), and growth of spinal interneuron neurites across mouse spinal injuries can be stimulated by treatments to enhance neuronal regenerative ability, astrocyte permissiveness and axonal chemoattraction (Anderson et al., 2016). Key components of neuroplasticity in these incomplete lesions are SpINs, as they form alternative routes to convey information between cells above and below the lesion (Courtine et al., 2008; May et al., 2017).

Here, we describe a robust model of long-term dissociated embryonic spinal cord cultures. During cultivation, neurons in these cultures form synapses, acquire mature electrical properties and markers of mature neurons, lose regenerative capacity, and express markers of SpINs. The culture model will be valuable for future developmental, functional as well as axonal regeneration studies.

## Materials and Methods

### Cell Culture

The method of generating mature spinal cord neurons was based on previously published methods for culturing spinal cord (Thomson et al., 2008), hippocampal and cortical neurons (Barbati et al., 2013; Koseki et al., 2017; Petrova et al., 2020), with modifications. Spinal neurons were isolated from E13.5-E14.5 embryos of the C57BL/6J mice. Spinal cords were dissected from embryos immersed in a cold Hibernate-E medium (Gibco, Thermo Fisher). The meninges were removed, and tissue was stored over ice in Hibernate-E (Gibco, Thermo Fisher). The collected tissue was washed with 1 ml of HBSS without Ca^2+^ or Mg^2+^ (Gibco, Thermo Fisher) two times. Next, the tissue was digested in papain-based Neuron Isolation Enzyme (Thermo Scientific™ Pierce) by adding 30 μl of enzyme solution per 1 spinal cord for 9 min at 37°C. After digestion, the enzyme was carefully removed, and the tissue was placed in disruption medium 1 (DM1) (Table 1). Tissue was disrupted by trituration with P1000 tip and left to settle for 2 min. The supernatant was transferred to a new tube, while the tissue pellet was triturated again in disruption medium 2 (DM2) (Table 1). This step was repeated once more if any tissue fragments remained after the second disruption. Supernatant containing disrupted tissue was transferred by a polished Pasteur pipette into a new falcon tube through a 40 μm cell strainer to remove undisrupted tissue fragments. Plating medium (PM) (Table 1) was added in a 1:1 ratio to the filtered solution containing the cells. The cell suspension was centrifuged for 5 min at 90 *g* at 37°C, the supernatant was removed, and the pellet was resuspended in 2 ml of PM. Then, the cells were counted (approximate expected amount is 1.2 million cells per spinal cord) and plated on glass coated with 100 μg/ml poly-D-lysine (Thermo Fisher) dissolved in pH 8.6 borate buffer. The cells were cultured either in a 24-well plate on sterilized 12 mm borosilicate glass coverslips (Karl Hecht), or glass-bottom 10-well CELLview™ Cell Culture Slides (Greiner). The optimal plating density of dissociated spinal cord cells in our cultures was around 93,000 cells/cm^2^. The best plating method that resulted in evenly distributed cells on the coverslips, was to dilute the dissociated cells in PM to a final concentration of 500,000 cells/ml and then add 350 μl of cell suspension into 24-well plate, or 80 μl into 10-well cell culture slide. After 1 h, most of the cells adhered to the coverslips, and cultivation medium (CM) was added to the PM in 1:1 proportion, but not before some of the PM was discarded. Final media volume used for cultivation in the 24-well plate was 500 μl and in the 10-well cell culture slide, it was 150 μl. Cultures were maintained by exchanging 1/2 of the media volume every 2 days. To limit the proliferation of glia, CM with the ITS+ supplement was replaced with CM without ITS+ after 7 days of culture. Importantly, we were able to maintain the cultures for 72 days.

**TABLE 1:** Media compositions used for preparation and maintaining spinal cord cultures.

Medium	Composition	Approximate total volume needed
Disruption medium 1 (DM1)	Hibernate-E (Gibco, Thermo Fisher), 0.8% BSA (Sigma-Aldrich) 100 μg/ml DNase (Sigma-Aldrich)	1 ml
Disruption medium 2 (DM2)	Hibernate-E, 0.4% BSA 20 μg/ml DNase	2 ml
Plating medium (PM)	50% DMEM, low glucose (Gibco, Thermo Fisher) 25% Horse serum (Gibco, Thermo Fisher) 25% HBSS without Ca^2+^ or Mg^2+^ (Gibco, Thermo Fisher) 1% Penicillin-Streptomycin (10,000 U/mL) (Gibco, Thermo Fisher)	30 ml
Cultivation medium (CM)	MACS Neuro Medium (Miltenyi Biotech) 2% NeuroBrew 21 (Miltenyi Biotech) 1% Glutamax (Thermo Fisher) *1% ITS+ (Sigma-Aldrich) 1% Penicillin-Streptomycin	50 ml

*ITS+ was excluded from cultivation media in cultures cultivated for more than 7 days.

### Plasmid Transfection

Cultures were transfected with CAG-GFP plasmid using NeuroMag magnetofection (OZ Biosciences) at DIV3. The CM was removed from the wells, stored, and replaced with 80% of regular cultivation volume of unsupplemented MACS Neuro Medium (Miltenyi Biotech). An appropriate amount of DNA plasmid was mixed with magnetic beads in OptiMEM medium (Gibco, Thermo Fisher) and incubated for 15 min at room temperature (RT), facilitating the binding reaction. The amount of reagents used for the transfection depended on the cultivation volume of the particular plate used in experiment. The proportions were calculated according to an optimized 24-well plate protocol, where 0.2 μg of DNA, 0.8 μl of magnetic beads, and 100 μl of OptiMEM were added per well. OptiMEM volume represented 20% of the regular cultivation volume. After the incubation, the mixture was added dropwise into wells with cells and unsupplemented medium. Plates were incubated on top of a strong magnet, purchased along with NeuroMag Starting Kit (OZ Biosciences), for 20 min at 37°C. The magnetic plate was removed and cells were incubated for another 40 min, after which original CM was returned to the wells. GFP was expressed in the culture 24 h after transfection. The conditions of transfection were optimized to attain low-efficacy transfection so that only a few of the cells in the culture expressed GFP and their morphology could be observed.

### Immunocytochemistry

Cells were fixed using 4% paraformaldehyde in phosphate-buffered saline (PBS) for 15 min at RT, washed twice, and kept in PBS at 4°C until further use. The staining protocol was started with permeabilization and blocking of the sample using solution containing 10% goat or donkey serum (according to secondary antibodies used) and 0.4% Triton-X in PBS for 1 h at RT with shaking. Next, primary antibodies were diluted in 2% goat serum and 0.1% Triton-X in PBS according to concentrations indicated in Table 2. Primary antibodies were incubated with the cells overnight at 4°C with gentle shaking, after which the solution was aspirated and the coverslips were washed twice with PBS. Secondary antibodies, diluted in the same solution as primary antibodies, were incubated with the samples for 1 h at RT. Next, nuclei were stained with DAPI (1/3,000 in PBS) for 5 min. Coverslips were then washed two times with PBS and mounted on microscopy slides with Aqua-Poly/Mount (Polysciences) and kept in the dark at 4°C until imaging.

**TABLE 2:** List of antibodies used for immunocytochemistry.

Primary antibodies					

**Immunogen**		**Dilution**		**Manufacturer**	**Cat. #**
Chx10		1/100		Santa Cruz	sc-365519
Doublecortin		1/200		Santa Cruz	sc-271390
GDNF Receptor alpha 1		1/100		Abcam	ab8026
Gephyrin		1/500		Synaptic systems	147111
GFP		1/1,000		Thermo Fisher	A10262
Homer 1		1/500		Synaptic systems	160003
Lbx1		1/10,000		Prof. Dr. Carmen Birchmeier-Kohler’s lab	
Lmx1b (guinea pig)		1/10,000		Prof. Dr. Carmen Birchmeier-Kohler’s lab	
Lmx1b (rabbit)		1/10,000		Prof. Dr. Carmen Birchmeier-Kohler’s lab	
Neurofilament 70 kDa		1/400		Sigma-Aldrich	MAB1615
Parvalbumin		1/500		Synaptic systems	195002
PAX2		1/200		Thermo Fisher	71–6,000
PKC γ		1/100		Santa Cruz	sc-166385
Tlx3 (guinea pig)		1/20,000		Prof. Dr. Carmen Birchmeier-Kohler’s lab	
Tlx3 (rabbit)		1/10,000		Prof. Dr. Carmen Birchmeier-Kohler’s lab	
VGAT		1/500		Synaptic systems	131008
VGLUT 1		1/500		Synaptic systems	135011
WFA		1/500		Sigma-Aldrich	L1516
βIII tubulin		1/1,000		Abcam	ab78078
βIII tubulin		1/1,200		Abcam	ab68193

**Secondary antibodies**					

**Immunogen**	**Fluorophore**		**Dilution**	**Manufacturer**	**Cat. #**

Chicken IgY	Alexa Fluor 488		1/200	Thermo Fisher	A-11039
Guinea Pig IgG (H + L)	Alexa Fluor 546		1/200	Thermo Fisher	A-11074
Mouse IgG (H + L)	Alexa Fluor 633		1/200	Thermo Fisher	A-21052
Mouse IgG (H + L)	Alexa Fluor 594		1/200	Thermo Fisher	A-11032
Mouse IgG (H + L)	Alexa Fluor 488		1/200	Thermo Fisher	A-11001
Rabbit IgG (H + L)	Alexa Fluor 594		1/200	Thermo Fisher	A-11012
Rabbit IgG (H + L)	Alexa Fluor 546		1/200	Thermo Fisher	A-11035
Rabbit IgG (H + L)	Alexa Fluor 488		1/200	Thermo Fisher	A-11034
Rabbit IgG (H + L)	Alexa Fluor 405		1/200	Thermo Fisher	A-31556

### Microscopy and Image Analysis

Brightfield images of live cultures were captured on a Zeiss Axio Vert.A1 inverted microscope equipped with AxioCam ERc 5s camera.

Fluorescence microscopy of the cultures was done on a LEICA CTR 6500 microscope. Analysis of neuronal and non-neuronal composition of the culture during cultivation was done by counting DAPI+ nuclei and DAPI+ nuclei colocalized with βIII-tubulin-positive cells with Fiji software (Schindelin et al., 2012). Expression of doublecortin (DBC) and neurofilament 70 kDa (NF70) during cultivation was analyzed by measuring the average gray value of captured fluorophore of a random region on coverslips using Fiji software. To define the background signal, the mean gray value was also measured in three random, smaller areas of the analyzed region without apparent signal. Morphological analysis of GFP-transfected neurons was performed using Fiji SNT plugin (Arshadi et al., 2020). Analyzed morphological metrics were: average length of branches, cable length, number of branches, axonal length, number of axonal branch points, length of axonal branches, average dendrite length, cable length of dendrites, and number of dendrites. Axons and dendrites were identified by morphologic norms established by Kaech and Banker (2006).

Confocal microscopy images were captured on a Zeiss LSM 880 Airyscan inverted microscope. Confocal images were used for presynaptic and postsynaptic marker colocalization analysis as well as spinal cord neuronal markers visualization. A Z-stack of images with 0.5 μm thickness increment was captured in random regions of the coverslip. Maximum frontal orthogonal projection of the Z-stack made in ZEN 3.1 (blue edition) was used for colocalization analysis. Synapses were counted using Puncta Analyzer v2.0, a Fiji plugin written by Bary Wark^1^.

### Laser Axotomy

Cells cultured on Greiner Bio-One CELLview™ Cell Culture Slides, transfected with GFP plasmid at DIV3, were kept in the microscope incubation chamber at 37°C and 5% CO_2_. After finding regions of interest, cells were captured before cutting using Carl Zeiss AxioObserver.Z1 microscope with confocal module LSM 880 NLO. Objective LD LCI Plan-Apochromat 25x/0.8 Imm Corr DIC M27 with oil immersion was used in the experiment. Next, axotomy was performed using a Ti: Sapphire femtosecond laser Chameleon Ultra II (Coherent), set at 900 nm. The cut was achieved by scanning a 3.4 μm long line across the axon, approximately 250 μm (253.8 ± 75.160 μm) from the cell body, in line-scan mode repeatedly 100–200 times, using 80–100% of the laser power. Axons were identified by morphologic norms established by Kaech and Banker (2006). Cells were then scanned every 30 min for 14 h to observe the post-axotomy response. Images were analyzed using ZEN 3.1 (blue edition) (Carl Zeiss Microscopy GmbH) and Fiji.

### Electrophysiological Recordings

The patch-clamp technique in the whole-cell configuration was used to evaluate the cell membrane properties of neurons. Micropipettes with a tip resistance of approximately 10 MΩ were made of borosilicate glass using a P-97 Flaming/Brown micropipette puller (Sutter Instruments) and filled with intracellular solution (0.5 mM CaCl_2_, 130 mM KCl, 2 mM MgCl_2_, 3 mM ATP, 5 mM EGTA, and 10 mM HEPES with pH 7.2). Coverslips with the cultures were placed on the recording chamber of an upright Axioskop microscope (Zeiss), equipped with a high-resolution AxioCam HR digital camera (Zeiss) and electronic micromanipulators (Luigs and Neumann). Electrophysiological data were recorded on cells perfused in artificial cerebrospinal fluid (2.7 mM KCl, 135 mM NaCl, 1 mM MgCl_2_, 2.5 mM CaCl_2_, 10 mM glucose, 1 mM Na_2_HPO_4_ and 10 mM HEPES with osmolality 312.5 ± 2.5 mOsmol/kg and pH 7.4). The signals were measured with a 10 kHz sample frequency and amplified with an EPC9 amplifier, controlled by the PatchMaster software (HEKA Elektronik), and filtered using a Bessel filter. Resting membrane potential (E_*m*_) was recorded by switching the EPC9 amplifier to the current-clamp mode. Raw data were processed with the FitMaster software (HEKA Elektronik). Input resistance (IR) was assessed from the current value at 40 ms after the onset of the 50 ms depolarizing pulse from the holding potential of −70 mV to −60 mV. Membrane capacitance (C_*m*_) was determined by Lock-in protocol in the PatchMaster software. To measure the sodium currents (I_*Na*+_), neurons were depolarized, and amplitude of the current was recorded at voltage step with the maximal current activation. In order to isolate the Na^+^ component only, the time- and voltage-independent currents were subtracted, and the peak value was considered the I_*Na*+_. Action potentials (AP) were recorded in the current-clamp mode. The current varied from 50 pA to 1 nA, in 50 pA increments; the pulse duration was 300 ms. Cells that produced at least one AP were considered capable of generating AP.

### Statistics

Statistically significant differences between multiple time points during culture cultivation groups were determined by Mann–Whitney test, one-way or two-way ANOVA, followed by Tukey’s multiple comparisons *post hoc* test (GraphPad Prism 9 software). Differences were regarded as significant at *p* < 0.05. Graphs were drawn using GraphPad Prism 9 software as means ± standard error of the mean (SEM), while the level of statistical significance was marked as follows: *p < 0.05, ^**^p < 0.01, ^***^p < 0.001.

## Results

The study aimed to establish culture conditions that would enable stable cultivation, maturation, and characterization of spinal cord interneurons isolated from E14 mice embryos. The composition of the culture during cultivation was analyzed using immunocytochemistry; maturity of the neurons was analyzed by immunocytochemistry and patch-clamp, and regenerative capacity of neurons was established by laser axotomy.

### Cellular Composition Changes During Cultivation

**FIGURE 1:**
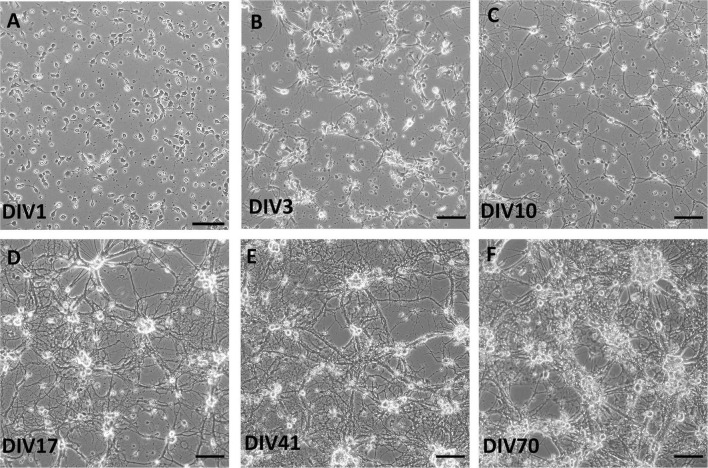
Brightfield images of live spinal cord culture in day *in vitro* (DIV) 1 **(A)**, DIV3 **(B)**, DIV10 **(C)**, DIV17 **(D)**, DIV41 **(E)**, and DIV70 **(F)**. Growth of processes was observed already at DIV3, while more complex network occurred in older cultures, after DIV17. Scale bars: 50 μm.

At day *in vitro* (DIV) 1, cells were attached and started to grow processes (Figure 1). Cells formed processes at DIV1, which extended during DIV3 and DIV10. Formation of complex network of processes over the whole culture surface could be seen at DIV17, and more robustly in older cultures at DIV41 and DIV70.

**FIGURE 2:**
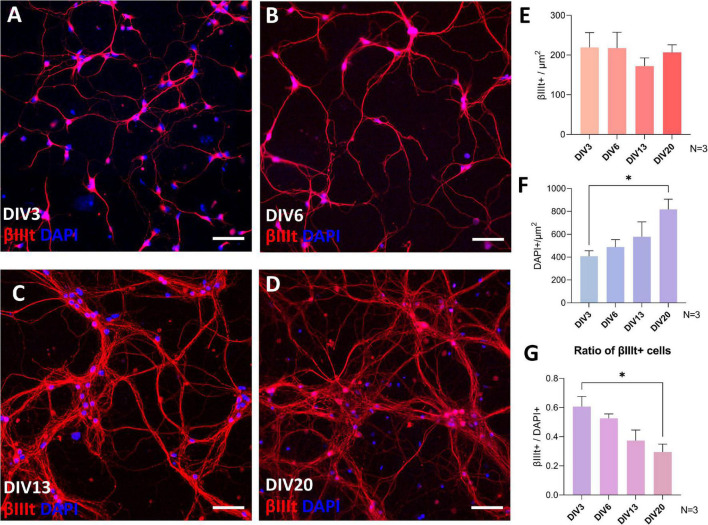
βIII tubulin and DAPI immunocytochemistry analysis of the cultures during day *in vitro* (DIV) 3 **(A)**, DIV6 **(B)**, DIV13 **(C)**, and DIV20 **(D)**. The number of neurons was stable during cultivation **(E)**, but the number of cells assessed by DAPI increased continuously **(F)**, which led to a decrease in the ratio of neuronal population in the culture **(G)**. Scale bars: 50 μm. Data are shown as means + SEM of *N* = 3 biological replicate cultures; *p < 0.05.

The total number of neurons in spinal cord culture was assessed by counting βIII-tubulin-positive cells per unit area of the fluorescence microscopy images (Figure 2). Although the total number of neurons remained stable (Figure 2E), the total number of cells, assessed by counting DAPI-stained nuclei in the culture, continued to rise during cultivation (Figure 2F). Due to the proliferation of glia, the neuronal fraction of the culture steadily, but significantly, declined from the average of 60.6% at DIV3 to 29.5% at DIV20 (Figure 2G). Despite the glial proliferation, long-term cultivation was achieved, with the longest maintained culture surviving past DIV72.

### Electrophysiological Properties of Neurons Mature by DIV16

To describe the maturation process of embryonic spinal cord culture on the functional level, electrophysiological properties of neurons were recorded using patch-clamp technique in the whole-cell configuration at four timepoints: DIV2, DIV9, DIV16, and DIV24.

**FIGURE 3:**
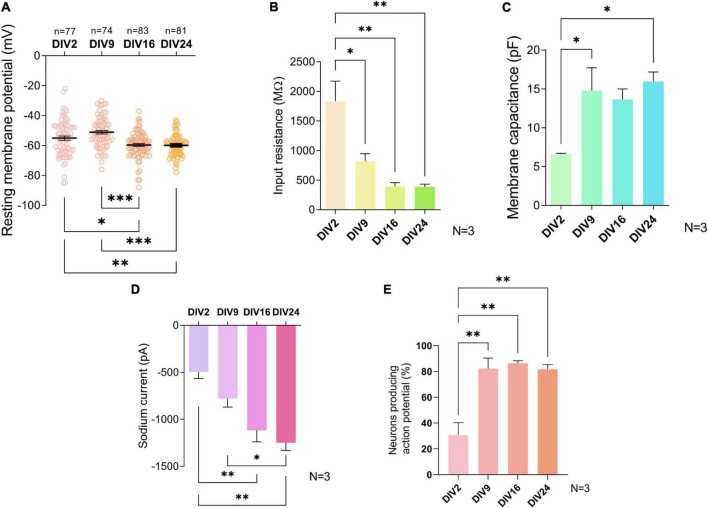
Electrophysiological properties of cultured neurons during maturation. Resting membrane potential **(A)** and sodium current **(D)** did not change significantly after day *in vitro* (DIV) 16. Input resistance **(B)**, membrane capacitance **(C)**, and the percentage of neurons producing action potentials **(E)** did not change significantly after DIV9. Data are presented as means ± SEM of *N* = 3 biological replicate cultures and n number of cells; *p < 0.05, ^**^p < 0.01, ^***^p < 0.001.

The E_*m*_ is considered a general characteristic of mature neurons (Sun et al., 2018). Average E_*m*_ of neurons significantly decreased from −55 ± 12.2 mV and −51.1 ± 10 mV at DIV2 and DIV9, respectively, to −59.6 ± 8.8 mV at DIV16 (*p* = 0.01 and *p* = 0.006) and to −59.9 ± 7.1 mV at DIV24 (*p* = 0.009 and *p* < 0.001) (Figure 3A), a figure consistent with mature interneurons. No further significant shift in E_*m*_ was observed between the neurons cultured for 16 and 24 days.

The IR is inversely proportional to the number of open ion channels and the size of the cell. A decrease in IR has been routinely used as an indicator of maturation of neurons in previous studies (Takazawa et al., 2012; Kopach et al., 2020). The average IR of studied neurons dropped significantly from 1,832 ± 591.7 MΩ at DIV2 to 817 ± 224.7 MΩ at DIV9 (*p* = 0.021) (Figure 3B). At later timepoints, IR values of 392.7 ± 111.1 MΩ at DIV16 and 387.3 ± 79.39 MΩ at DIV 24 were not significantly decreased, compared to IR at DIV9.

Cell size increased during development due to growth of the cells, which is why total C_*m*_, a physical quantity directly proportional to the membrane surface area, is a useful tool to assess changes in neuronal maturation in culture (Golowasch et al., 2009). We found that a statistically significant shift in C_*m*_ value occurred between DIV2 and DIV9, after which no significant changes ensued (Figure 3C). At DIV2, the average C_*m*_ was 6.63 ± 0.13 pF while at DIV9, it was 14.77 ± 5.09 pF (*p* = 0.042).

The I_*Na*+_ plays a significant role in the action potential amplitude and has been reported to change during differentiation of embryonic and human-induced pluripotent stem cells into neuronal cells (Song et al., 2013). At DIV9, neurons in our culture exhibited an average I_*Na*+_ of −785.4 ± 387.5 pA, which was significantly lower compared to DIV24 I_*Na*+_ of −1,248 ± 684.3 pA (*p* = 0.031) (Figure 3D).

Neurons in primary cultures were reported to have little spontaneous activity in the initial stages of cultivation, however, they exhibit it in later stages during cultivation, corresponding with synapse formation (Norris et al., 2006). The fraction of neurons generating AP increased significantly between DIV2 and DIV9 (*p* = 0.003) (Figure 3E) and did not change significantly in more mature cultures.

### Maturity Markers of Primary Cortical Cultures Are Regulated in Primary Spinal Cord Cultures

**FIGURE 4:**
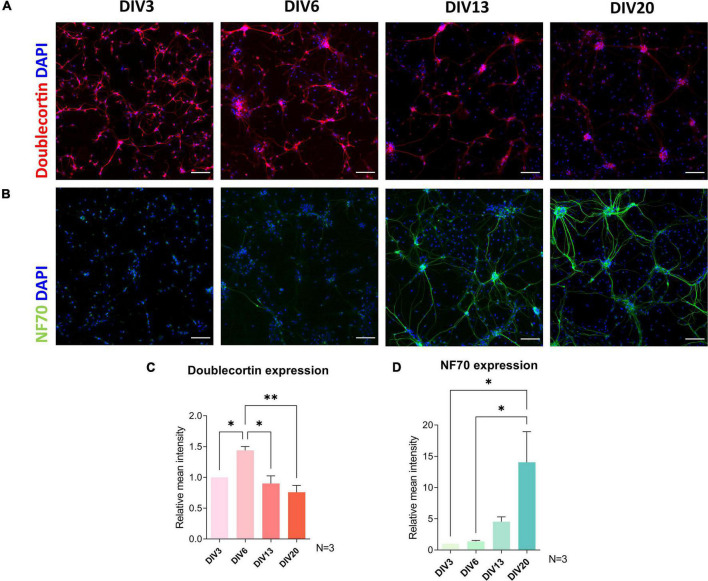
Immunocytochemical analysis of maturity markers doublecortin **(A,C)** and neurofilament 70 kDa (NF70) **(B,D)** expression during cultivation. Doublecortin expression was downregulated after DIV6, while a significant increase in NF70 signal was observed after DIV13. Scale bars: 100 μm. Data are presented as means + SEM of *N* = 3 biological replicate cultures; *p < 0.05, ^**^p < 0.01.

Koseki et al. (2017) identified maturity markers in embryonic cortical neuron *in vitro* model by RNA sequencing and confirmed by immunocytochemistry. NF70 is upregulated, while DBC is downregulated during maturation of these cultures. To assess the maturation process of spinal cord cultures, we analyzed expression of above-mentioned markers using immunocytochemistry (Figure 4). We found that the greatest downregulation of DBC expression in our cultures was between DIV6 and DIV13 (*p* = 0.011). NF70 immunoreactivity increased most significantly at DIV20, compared to DIV3 (*p* = 0.025) and DIV6 (*p* = 0.029).

### Cells in the Spinal Cord Culture Form Inhibitory and Excitatory Synapses

**FIGURE 5:**
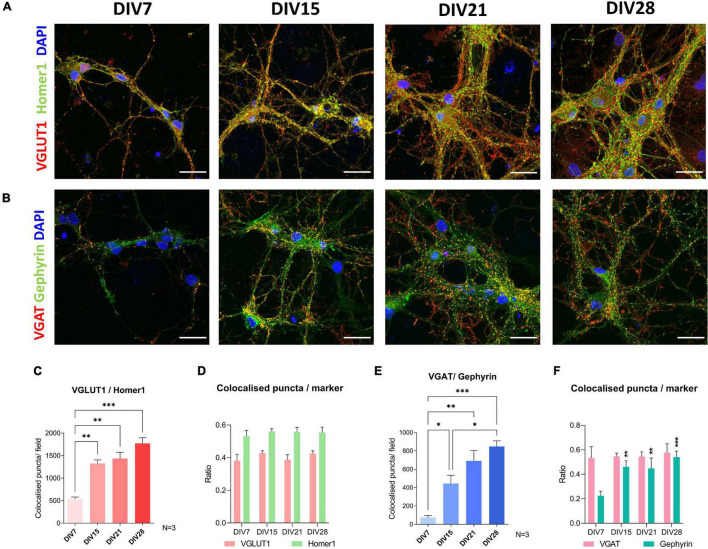
Immunocytochemical analysis of synapse formation during cultivation of spinal cord culture. For excitatory synapse analysis, colocalization of VGLUT1 and Homer1 was examined **(A,C,D)**, while colocalization of synaptic markers VGAT and Gephyrin was used for analysis of inhibitory synapses **(B,E,F)**. Formation of new excitatory synapses was the most prominent between day *in vitro* (DIV) 7 and 15 **(C)**. A similar pattern was observed in colocalization analysis of inhibitory synapses **(E)**. VGLUT1, Homer1, and VGAT colocalization ratios were stable during cultivation **(D,F)**, while an increase of ratio of Gephyrin marked synapses that colocalized with VGAT occurred between DIV7 and DIV15 **(F)**. Scale bars: 25 μm. Data are presented as means + SEM of *N* = 3 biological replicate cultures; *p < 0.05, ^**^p < 0.01, ^***^p < 0.001.

To investigate neuronal connectivity and network development in the culture, we investigated colocalization of pre-synaptic and post-synaptic markers using immunocytochemistry (Figure 5). The excitatory pre-synaptic marker, vesicular glutamate transporter 1 (VGLUT1), was colocalized with Homer1, a post-synaptic density protein, that has a role in directing glutamate receptors. The largest shift in colocalization of these two markers was detected between DIV7 and DIV15 (*p* = 0.003) (Figure 5C). After DIV15, we saw no significant increase in the colocalization of excitatory synapse markers. Regarding inhibitory synapses, colocalization of vesicular GABA transporter (VGAT) and gephyrin, a scaffold protein responsible for shaping the inhibitory postsynaptic density, was analyzed (Figure 5E). Similar to excitatory synapses, inhibitory synaptic marker colocalization increased significantly between DIV7 and DIV15 (*p* = 0.043). However, we also detected a significant increase in inhibitory synapse numbers between DIV15 and DIV28 (*p* = 0.029).

To assess whether the individual synaptic markers colocalize to differing extents during maturation, we analyzed the average ratio of co-localized synaptic puncta compared to total puncta counted for each individual synaptic marker (Figures 5D,F). We found that during cultivation, VGLUT1, Homer1, and VGAT colocalized in similar ratios, meaning that in immature and mature cultures, the same fraction of these synaptic markers did not colocalize with their counterpart synaptic marker. On the other hand, the gephyrin co-localized fraction was not as stable (Figure 5F). We detected an increase in the colocalized gephyrin fraction between DIV7 and DIV15 (*p* = 0.005).

### Diverse Neuronal Markers Are Expressed in Primary Spinal Cord Culture

**FIGURE 6:**
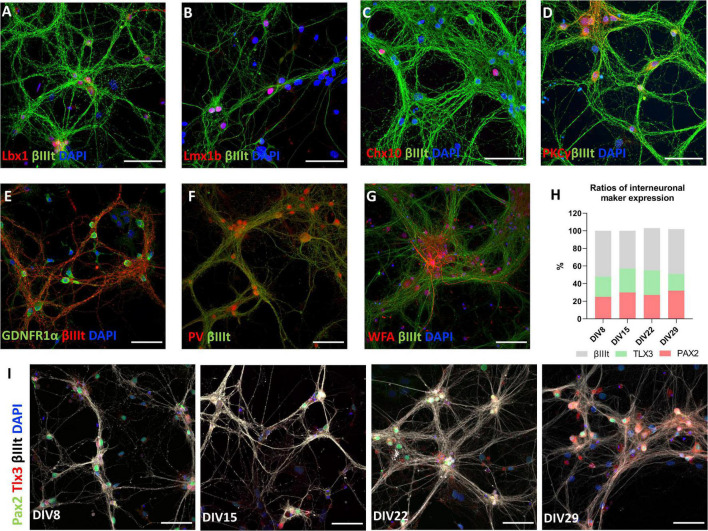
Markers of spinal interneurons Lbx1 **(A)**, Lmx1b **(B)**, Chx10 **(C)**, Pax2, and Tlx3 **(I)** are present in primary spinal cord cultures. Pax2+ and Tlx3+ cells represent approximately half of neurons in the culture **(H)**. Neurons also expressed PKCγ **(D)**, GDNFR1α **(E)**, parvalbumin (PV) **(F)**, and *Wisteria floribunda* agglutinin (WFA) **(G)**. Scale bars: 50 μm.

Utilizing immunocytochemistry in mature cultures, we confirmed that neurons express transcription factors associated with particular types of spinal interneuron. Lbx1 (Figure 6A), Lmbx1 (Figure 6B), Chx10 (Figure 6C), Tlx3, and Pax2 (Figure 6I) were expressed in differing proportions of neurons in our *in vitro* model. These transcription factors have been used as markers of neuronal classes in developing spinal cord (Alaynick et al., 2011; Lu et al., 2015). Counting the number of neurons stained by these markers, the most frequent markers in the culture were Pax2 and Tlx3. By counting Tlx3+ and Pax2+ nuclei of neurons identified by βIIItubulin staining, we observed that Pax2 is expressed by approximately 28.5% of neurons and Tlx3+ by 24.3% of neurons in the culture on average and these values did not change significantly during cultivation (Figure 6H). Coexpression of both markers by the same cell was almost non-existent and the ratio of expression of both markers did not change significantly during maturation. Similarly, we estimated that 5.8% of neurons were Lbx1+ and 35.3% of neurons were Lmx1b+ at DIV17. Chx10+ neurons were sparsely present in the culture at DIV20, on average 30 cells per coverslip. Some motoneurons identified by ChAT staining were present in the culture in the first few days of the culture (data not shown), however, none of them survived during long-term cultivation. Next, we confirmed that subsets of neurons in the culture expressed protein kinase C gamma (PKCγ) (Figure 6D), parvalbumin (PV) (Figure 6F) at DIV20, and GDNF family receptor alpha-1 (GFRα1) at DIV 17 (Figure 6E). These three proteins were expressed by the majority of neurons in the culture. *Wisteria floribunda* agglutinin (WFA) staining around neuronal cell bodies and dendrites identifies perineuronal nets (PNNs). Neurons expressing perineuronal nets were only observed in exceptionally aged cultures (Figure 6G). At DIV72, we observed on average 23.5 WFA+ neurons per coverslip.

### Neurons in Primary Culture Have Distinct Morphologies

**FIGURE 7:**
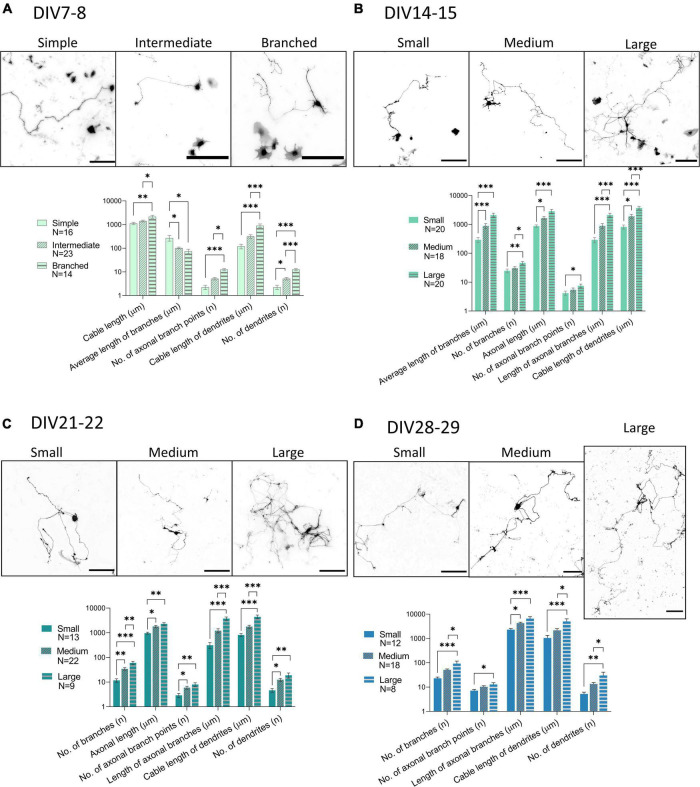
Morphological groups of neurons transfected with GFP (black) during various days *in vitro* (DIV), DIV7-8 **(A)**, DIV14-15 **(B)**, DIV21-22 **(C)**, DIV28-29 **(D)**. While DIV7 neurons were successfully categorized according to the number of processes into simple, intermediate, and branched, from DIV14 onward, the length of processes was found to be a better parameter for segregating morphologies into small, medium, and large. Scale bars: 200 μm. One-way ANOVA with Turkey’s *post hoc* test was used for analyzing the difference between morphological groups. Data are presented as means + SEM of N number of cells from 3 biological replicates; *p < 0.05, ^**^p < 0.01, ^***^p < 0.001.

To investigate the morphology of neurons present in the culture, we transfected the cultures with GFP at DIV3 using a low transfection efficiency protocol. This resulted in expression of GFP by only a few cells on the coverslip, whose morphology could then be studied. The number and length of processes and their branches were analyzed with the SNT plugin in Fiji. DIV7-8 cells were sorted into three groups, simple, intermediate, and branched, based on the number of processes (Figure 7A and Table 3). Successful separation of the groups was confirmed by significant differences of multiple morphological parameters between the groups. In older cultures, at DIV14-15 (Figure 7B), DIV21-22 (Figure 7C), and DIV28-29 (Figure 7D), cable length (sum length of all analyzed processes) was identified as a better parameter for segregating morphologies. Morphologies identified in these timepoints were named small, medium, and large, but in all three DIVs, the range of cable length in each group was slightly different (Table 3).

**TABLE 3:** Morphological groups of neurons identified during cultivation of spinal cord cultures.

	Morphological group	Range	*N*	*N* total
DIV7-8	Simple	1–9 b	16	53
	Intermediate	10–19 b	23	
	Branched	20–58 b	14	
DIV15-16	Small	300–3,000 μm	20	58
	Medium	3,000–6,000 μm	18	
	Large	6,000–13,000 μm	20	
DIV21-22	Small	900–3,000 μm	13	44
	Medium	3,000–7,500 μm	22	
	Large	7,500–14,000 μm	9	
DIV28-29	Small	1,700–4,500 μm	12	38
	Medium	4,500–8,000 μm	18	
	Large	8,000–20,000 μm	8	

*DIV7-8 neurons were separated according to the number of branches (b), while older neurons were grouped according to the sum length of their processes.*

In an effort to identify neuronal subtypes that these distinct morphologies belong to, we costained the GFP expressing neuronal cultures for Pax2 and Tlx3 (data not shown). We chose these markers because they were expressed by a large portion of neurons in the culture and because they allowed us to identify two different types of interneurons. We found that there were no clear differences between the morphologies of Pax2+ and Tlx3+ neurons at any of the analyzed time points of cultivation.

**FIGURE 8:**
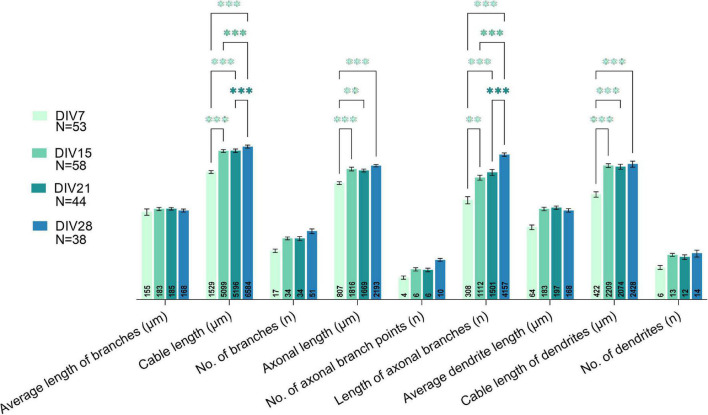
Comparison of all studied morphological parameters between various days *in vitro* (DIVs). A major shift in morphological properties was observed between DIV7 and DIV15. Growth of processes was observed even in prolonged cultivation periods, as parameters related to length of processes increased significantly at DIV28. Two-way ANOVA followed by Turkey’s *post hoc* test was used for analyzing the difference between DIVs. Data are presented as means ± SEM, means of plotted values are annotated in each bar; ^**^p < 0.01, ^***^p < 0.001 of N number of cells from 3 biological replicates.

Comparison of morphological parameters of all neurons at different maturation stages revealed marked changes in the morphology of the neurons between DIV7-8 and DIV14-15 (Figure 8). The majority of the studied parameters did not significantly change after DIV15, except for the sum of all processes (cable length) and the sum of axonal branches. This indicates that even in older cultures, neuronal processes continue to grow.

### Regenerative Capacity of Axons Decreases With Maturity

**FIGURE 9:**
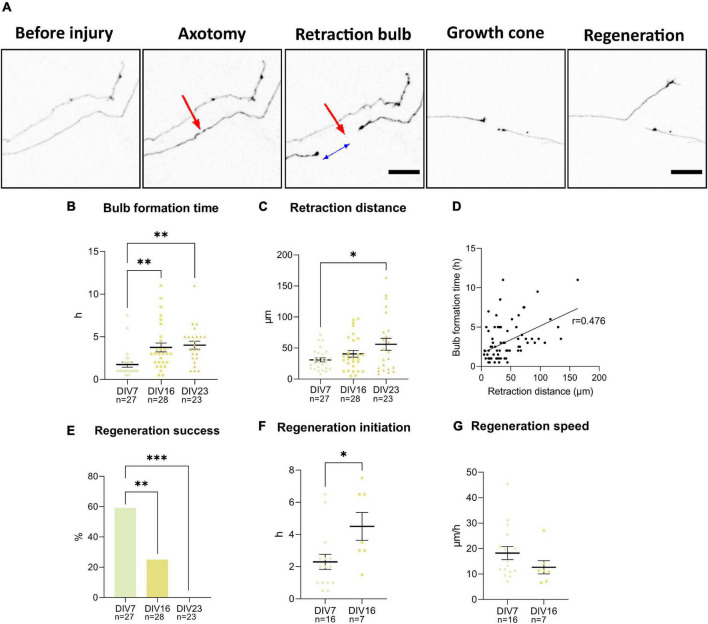
Axotomy of the GFP (black) transfected neurons was achieved using a 900 nm laser **(A)**. Red arrow points at the location of the cut. Retraction bulb formed at certain distance from the injury site as indicated by the blue arrow. Bulb formation time **(B)** and retraction distance **(C)** were lower in DIV7 neurons. These two parameters have a positive linear relationship demonstrated by Pearson’s correlation coefficient **(D)**. Percentage of regenerating axons decreased significantly between DIV7 and DIV16 **(E)**. Time between retraction bulb formation and growth cone formation increased significantly at DIV16 **(F)**, while speed of regeneration decreased marginally **(G)**. Scale bars: 25 μm. Data are presented as means ± SEM of n number of cells from 3 biological replicates; *p < 0.05, ^**^p < 0.01, ^***^p < 0.001.

To characterize the regenerative capacity of neurons in the cultures, axons of individual cells at DIV7, 16, and 23 were cut using a 900 nm laser and observed over 14 h. Individual cells and their processes were visualized utilizing low-efficiency GFP transfection at DIV3. After the cut, cells either died or managed to close the damaged area and form a characteristic retraction bulb—a swollen structure at the tip of the axon still attached to the cell (Figure 9A). The maturity of neurons did not affect the percentage of dead cells following the injury, which varied between 6–25% across individual experiments.

Formation of the retraction bulb sometimes occurred right next to the site of injury, or there was more retraction, and the retraction bulb formed further up the axons (Figure 9F). The time needed for formation of the retraction bulb was also variable. These two parameters—retraction distance and bulb formation time—were found to be significantly different in young neurons at DIV7, compared to DIV16 and DIV23 neurons (Figures 9B,C). In DIV7 neurons retraction distance was 31 ± 16.81 μm, while the bulb formation time was on average 1.7 ± 1.6 h. At DIV16, retraction distance was longer, at 40.6 ± 28.1 μm, while bulb formation time of 3.8 ± 2.7 h was prolonged significantly (*p* = 0.004). DIV23 neurons had both significantly longer retraction distance (56.1 ± 43.5 μm) and bulb formation time (4 ± 2.2 h), compared to DIV7 (*p* = 0.02 and *p* = 0.002). Correlation analysis of all cells at all time points revealed a positive correlation between retraction distance and bulb formation time (*p* < 0.001) (Figure 9D), which indicates that longer retraction leads to slower sealing of the axon tip.

After successful bulb formation, the axons either initiated regeneration by forming a growth cone or failed to regenerate. Failure of regeneration increased with maturity of the culture (Figure 9E). While at DIV7, 59.3% of axotomized neurons regenerated, only 25% regenerated at DIV16; no regenerating axons were observed at DIV23. In regenerating axons, initiation time, the time between retraction bulb and growth cone formation, was also evaluated (Figure 9F). We observed that regenerating DIV16 cells had longer initiation time compared to DIV7 neurons (*p* = 0.02). The speed of regeneration (length of newly synthesized axon in 2 h after initiation time) was lower at DIV7, although not significantly (Figure 9G).

## Discussion

In the present study, we optimized a method of culturing primary spinal cord neurons, investigated their growth and maturation, and tested their regenerative ability. The cultures are robust, able to survive long term (>2 months), and are maintained in defined, simple serum-free medium. With increasing time in culture, we observed proliferation of glia. The use of anti-mitotic agents, such as 5-Fluoro-2′-deoxyuridine (FdU) and arabinosylcytosine C (AraC) has been routinely used previously in primary neuronal cultures to eradicate dividing cells (Stahl et al., 2007; Roppongi et al., 2017). However, their use in long-term cultivation has been reported to be problematic. It can lead to death of neurons through glutamate excitotoxicity (Ahlemeyer et al., 2003), oxidative stress (Geller et al., 2001), or neurotrophic factor deprivation (Martin et al., 1990). On top of that, glial cells have been shown to be a crucial factor in synaptogenesis and in recreating complex functionality of the CNS in an *in vitro* system (Allen and Barres, 2005; Hui et al., 2016; Enright et al., 2020). For these reasons, we excluded the use of anti-mitotic agents in our culture, which led to proliferation of glia but did not affect the survival of neurons and probably encouraged neuronal maturation (Figure 2).

Our goal was to create a culture of spinal cord neurons that models the maturing and mature CNS and which is suitable for future experiments designed to enhance the ability of the neurons to regenerate. The viability of embryonic neuronal cultures is a great advantage in culture preparation, but the immaturity of the neurons after seeding is a problem as they display entirely different properties compared to mature neurons that have lost their intrinsic ability to regenerate (LaBarbera et al., 2021). To evaluate the maturity of our cultures, we investigated the expression of DBC, NF70, synaptic development, and electrophysiological properties of neurons during cultivation.

The alterations in the electrophysiological properties are hallmarks of neurodevelopment of spinal cord neurons (Durand et al., 2015) and have been used to stage neuronal maturation in cortical cultures (Koseki et al., 2017). The electrophysiological properties of neurons in our culture showed the typical changes associated with maturation until DIV16 after which there was no further change (Figure 3). In more detail, the major shift in most parameters in our functional studies already transpired before DIV9. Comparing these parameters to the electrical activity of neurons in P6-10 spinal cord slices recorded in a previous study by Sun and Harrington (2019), it can be argued that our neuronal culture is composed solely of interneurons. Interneurons in the cited study had high IR (305 ± 33 MΩ), E_*m*_ of −54.2 ± 1 mV, and 65% of them exhibited spontaneous activity, which helped in distinguishing interneurons from motoneurons. Actual postnatal maturity of the neurons in the culture is difficult to assess using electrophysiological properties. We compared the properties in our cells with spinal cord slice recordings of P6-14 mice (Wilson et al., 2005; Sun and Harrington, 2019) and P0-3 whole cord recordings (Zhong et al., 2006). While some parameters, such as E_*m*_, were comparable to neurons of P0-3 spinal cord, other properties such as IR and percentage of spontaneously firing neurons indicated that our neurons were more mature at DIV16 than those of the P4-16 spinal cords. These results suggest that our cultures at around DIV16 exhibit electrical properties of early to late postnatal neurons.

DBC is a microtubule-associated protein expressed by migrating neurons during development. It has been used as a marker of neuronal precursors (Ayanlaja et al., 2017), while its downregulation has been reported in mature neurons (Brown et al., 2003). Developmental regulation of DBC was also observed *in vivo*. In mouse embryonic extracts, DBC expression is absent at E10.5, but present from E12.5 to newborn. Although DBC expression is still present in neonatal mouse brain, it is absent in adult mice (Francis et al., 1999). We observed a significant decrease in DBC immunoreactivity after DIV6 of culture (Figure 4). NF70 has been previously used as a mature axonal marker (Lu et al., 2017). NF70 mRNA analysis in embryonic and early postnatal mouse spinal cords shows, that this transcript is present already in E13. The expression is downregulated until E18 and then starts to increase until postnatal day 21 (Pernas-Alonso et al., 1996). In our cultures, there was a sharp upregulation of this marker at DIV20. Maturity of the culture was also confirmed by observing the formation of neural networks (Figure 5). The number of excitatory and inhibitory synapses was increasing during the first 2 weeks of culture, after which they plateaued, which was previously observed to occur in cultures of primary cortical and striatal neurons between DIV14 and DIV21 (Moutaux et al., 2018).

Although there is no consensus of neuronal subtypes classification in adult spinal cord, there have been several recent studies that tackled this problem in various approaches (Zeng and Sanes, 2017; Hayashi et al., 2018; Dobrott et al., 2019). The most efficient way is to classify cell types by molecular markers- proteins expressed by only specific cell groups. Transcription factors have been used to classify spinal neurons, as they control neuronal specialization during development (Lu et al., 2015; Russ et al., 2021). Although the expression of the transcription factors can be transient- their expression is decreased after differentiation (Gosgnach et al., 2017), some continue to be expressed during adulthood (Del Barrio et al., 2013). We identified expression of several of these transcription factors in our cultures, namely Lbx1, Lmx1b, Chx10, Pax2, and Tlx3 (Figure 6). Lbx1 transcription factor participates in differentiation of dorsal horn neurons (Müller et al., 2002), it is expressed by laminae III-IV neurons of adult spinal cord, which are mostly excitatory, but not exclusively. Lmx1b+ neurons also express Lbx1, but they are only excitatory and expressed at high levels in laminae I-III (Del Barrio et al., 2013). Lmx1b+ neurons in our cultures were colocalized with majority of Tlx3 neurons (data not shown), which was also previously confirmed *in vivo* (Dai et al., 2008). Tlx3 transcription factor, present mostly in laminae I-II, has been identified to have direct control in specifying excitatory neurons in the dorsal horn (Monteiro et al., 2021). Apart from the previously mentioned, mostly excitatory, interneurons, we have also observed a large population of Pax2+ neurons in our cultures. Pax2 is essential for the differentiation of GABAergic neurons and its expression has been used as a marker of inhibitory neurons in the mouse dorsal horn (Larsson, 2017). The presence of ventral interneurons was also confirmed using Chx10 staining. Chx10 has been used as a marker of V2a excitatory interneurons in the ventral spinal cord, which participate in control of the limbs (Hayashi et al., 2018). In addition to transcription factors, other proteins have also been used to classify neurons in adult spinal cord. PKCγ is one such protein that is expressed by excitatory interneurons in lamina II that participate in mechanical and thermal allodynia (Neumann et al., 2008). Parvalbumin, on the other hand, is a marker of inhibitory interneurons, both GABAergic and glycinergic, located between the lamina II and III, which act as filters of low-threshold mechanoreceptive inputs (Petitjean et al., 2015; Häring et al., 2018). Both PKCγ and PV were expressed by our neuronal cultures. We found that ChAT positive cells do not survive long in the described culture. Motor neurons are known to be particularly vulnerable and rely heavily on trophic support of peripherally connected cells, such as muscle cells and Schwann cells for survival (Bucchia et al., 2018). The loss of trophic support after dissection could have eventually led to death of motor neurons in our cultures. Due to the lack of specific markers, we can only speculate if projection neurons shared the same fate. Projection neurons represent a very small portion (<1%) of neurons of the dorsal horn. They are located in lamina I and dispersed throughout lamina III–VI (Abraira and Ginty, 2013). The majority of projection neurons retrogradely traced by cholera toxin B subunit (CTb) were found to express neurokinin 1 receptor (NK1r) (Spike et al., 2003; Cameron et al., 2015), but a big portion of lamina I, IV, V and VI neurons, therefore mostly interneurons, express this receptor as well (Todd et al., 1998). NK1r immunostaining alone is therefore not sufficient to prove the presence of projection neurons.

To analyze the morphology of neurons during maturation and to attempt to classify them into characteristic groups, we measured the length and number of their processes. Previous studies classified the spinal cord neuron morphology according to laminar location, particular geometry, and neurite orientation (Grudt and Perl, 2002; Hantman et al., 2004), which is not possible *in vitro.* Gertz et al. (2010) reported increasing length and number of neurites of spinal cord motoneurons during cultivation, but 48 h. We analyzed the morphology parameters in the course of 4 weeks *in vitro*, during which we distinguished individual morphologies and observed growth of processes even in older cultures. The morphological groups that were identified were not characteristic of either Tlx3+ or Pax2+ cells, which are markers of two different interneuronal subtypes. Electrophysiological characteristics of the morphological groups were not assessed. Additionally, we observed that even in older cultures, neuronal processes continue to grow (Figure 8). Electrophysiological parameters IR and C_*m*_ correlate with cell size (Sun et al., 2018), but we did not see changes in these parameters after DIV9 (Figures 3B,C). It is important to note, that the morphological changes in older cultures were identified at DIV28, while the oldest cultures recorded by patch-clamp were at DIV24, which could be the reason for this inconsistency. On the other hand, we observed an increase in the number of synapses at DIV28 (Figure 5), which could indicate that elongation of the processes observed at the same timepoint in morphology analysis is due to formation of new connections between neurons in the culture.

By following events that occurred after axotomy of spinal cord neurons, we observed that more mature cells react more slowly to laser-induced injury and retract further from the injury site compared to younger cells (Figure 9). By DIV23, neurons lost all regenerative capacity and even at DIV16, the axon regeneration was slower. These results indicate that spinal neurons lose their regenerative capacity during maturation *in vitro*, similarly to cortical cultures, as was shown before (Koseki et al., 2017). We observed a similar transition in neuronal properties in other experiments around this time point as well. The major shift in morphology during *in vitro* maturation was observed between DIV7 and DIV15 (Figure 8). The same cultivation period was critical in changes of maturity marker expressions, synaptic connectivity, and most of the electrophysiological properties. These results indicate that major intrinsic maturation events are occurring at this time point in the cultured neurons. Loss of plasticity and regenerative ability in CNS neurons during development *in vivo* is well established (Fawcett, 2020). Apart from the inhibitory environment that is created at the injury site, mature neurons themselves lack intrinsic regenerative properties. During the loss of regenerative ability, neurons show changes in expression of growth-related molecules, and they also become polarized into somatodendritic and axonal domains. Axons alone lack molecules such as growth-related receptors, as well as mechanisms deemed vital for regeneration (Kappagantula et al., 2014; Franssen et al., 2015; Cheah et al., 2016). Apart from non-regenerating axons, CNS neurons do not express the regenerative program that is seen with the upregulation of many genes that is initiated after injury of peripheral nerves (Ben-Yaakov et al., 2012).

In conclusion, we developed, validated, and described a new culture of spinal cord neurons. We believe that the described culture efficiently models the biology of the spinal cord, which makes it a valuable tool for future studies.

## Data Availability Statement

The original contributions presented in the study are included in the article/supplementary material, further inquiries can be directed to the corresponding author/s.

## Ethics Statement

All cell isolations from mice embryos were performed in accordance with the European Communities Council Directive of 22.09.2010 (2010/63/EU) regarding the use of animals in research and were approved by the Ethics Committee of the Institute of Experimental Medicine ASCR, Prague, Czechia under no. 54/2017 (approved 14.10.2017 and valid till 31.07.2022).

## Author Contributions

IV drafted the manuscript, performed and analyzed all experiments, except patch-clamp recordings, which were performed by JK. JCK, JF, and PJ conceived of the project and supervised the experiments. All authors contributed to editing the manuscript.

## Conflict of Interest

The authors declare that the research was conducted in the absence of any commercial or financial relationships that could be construed as a potential conflict of interest.

## Publisher’s Note

All claims expressed in this article are solely those of the authors and do not necessarily represent those of their affiliated organizations, or those of the publisher, the editors and the reviewers. Any product that may be evaluated in this article, or claim that may be made by its manufacturer, is not guaranteed or endorsed by the publisher.

## Footnotes

1
https://github.com/physion/puncta-analyzer
